# Individual Anthropometric, Aerobic Capacity and Demographic Characteristics as Predictors of Heat Intolerance in Military Populations

**DOI:** 10.3390/medicina57020173

**Published:** 2021-02-17

**Authors:** Faith O. Alele, Bunmi S. Malau-Aduli, Aduli E. O. Malau-Aduli, Melissa J. Crowe

**Affiliations:** 1 College of Healthcare Sciences, James Cook University, Townsville, QLD 4811, Australia; 2 College of Medicine and Dentistry, James Cook University, Townsville, QLD 4811, Australia; bunmi.malauaduli@jcu.edu.au; 3 College of Public Health, Medical and Veterinary Sciences, James Cook University, Townsville, QLD 4811, Australia; aduli.malauaduli@jcu.edu.au; 4 Division of Tropical Health and Medicine, James Cook University, Townsville, QLD 4811, Australia; melissa.crowe@jcu.edu.au

**Keywords:** heat tolerance, aerobic capacity, hydration status, body composition, exertional heat illness, military, regression analysis, prediction

## Abstract

*Background and objectives*: The Australian Defence Force (ADF) engages in combat-related activities in hot climatic conditions, which exposes ADF members to the threat of exertional heat illness (EHI). After an episode of EHI, the heat tolerance test (HTT) is conducted to determine heat tolerance. Heat intolerance is the inability to maintain thermal balance while exercising in a hot environment. This study investigated the predictive roles of individual characteristics (age, gender, aerobic capacity (VO_2_max) and body composition) on physiological responses to the HTT in a group comprising ADF personnel and civilian volunteers. *Materials and Methods*: A quasi-experimental design was used and 52 (38 males and 14 females) participants were recruited from the ADF and the general population for the HTT. Heat intolerance was defined following the standard criteria for the HTT (temperature and heart rate). Data were analysed using inferential statistics. *Results*: The mean age of the participants was 31.1 ± 11.6 years, and 44% (23 people: 19 males and 4 females) of the participants were heat intolerant. Independent samples T-test showed that body mass index (*p* = 0.011) and body fat% (*p* = 0.034) of heat-intolerant participants were significantly higher than their heat-tolerant counterparts. Body surface area to mass ratio (*p* = 0.005) and aerobic capacity (*p* = 0.001) were significantly lower in heat-intolerant participants. Regression analyses showed that age, gender, aerobic capacity and body fat% were significant (*p* < 0.001) predictors of heat tolerance outcomes, with R^2^ values ranging from 0.505 to 0.636. *Conclusions:* This study showed that aerobic capacity, body fat%, age and gender are predictors of heat intolerance among military and non-military populations. However, there may be a need for future studies to consider identifying other indicators such as clinical biomarkers of heat intolerance, which could be used to develop a more reliable HTT protocol.

## 1. Introduction

The Australian Defence Force (ADF) is involved in combat-related activities in hot climatic conditions which exposes ADF members to the constant threat of exertional heat illness (EHI) [1]. The threat of EHI is significant, with heat stroke (a severe form of EHI) identified as the third most common combat-related injury (4.90% of the injuries) among ADF members between 2012 and 2014 [2]. Evidence suggests that EHI may cause acute loss of manpower, interruption of training and possible medical discharge (dismissal) from service if profound clinical sequelae such as multi-organ damage occur [3]. Therefore, individuals who are recruited into the military should have the physical and physiological capacities to tolerate heat stress [4]. However, individuals vary in their ability to tolerate heat stress and the inability to maintain thermal balance while exercising in a hot environment is known as heat intolerance [5,6,7]. Previous studies have reported that heat intolerance may be a by-product of inherent impaired thermoregulatory mechanisms or may occur as a direct result of exertional heat stroke (EHS) [8,9].

As part of the strategies to manage heat illness, the ADF developed and introduced a policy to treat heat casualties [10,11]. ADF members who have experienced a known or suspected case of heat stroke are required to undergo the standard heat tolerance test as part of the return-to-duty process [11]. The standard heat tolerance test (HTT) used by the ADF is the test developed by the Israeli Defence Force in 1979 as part of the return-to-duty process for military personnel who have suffered EHS [12,13]. While the test is being used by some military populations as part of the return-to-duty process, the use of the cut-off values is debatable [14]. The HTT is considered as a functional test that is reflective of the current state and may not be predictive of future injury given that there are other individual and environmental factors that may contribute to the risk [14]. Although the HTT may help with decision-making processes to determine individual responses to heat stress, less is known about the predictors of heat intolerance, particularly in military populations [15].

Only a few studies have attempted to identify the factors that predict heat intolerance in military populations [16,17]. From these studies, it has been established that low aerobic fitness is associated with heat intolerance [16,17]. In addition, low aerobic capacity was associated with the incidence of EHI in military populations [18,19,20]. Other individual factors such as age, gender and body composition including body fat percentage (BF%), body mass index (BMI), body surface area (BSA) and body surface area to mass ratio (BSA/M ratio) have been reported to influence heat tolerance [16,21,22,23]. For example, differences in BSA/M ratio, BF%, sweat rate and aerobic capacity account for the variation in heat tolerance between men and women [16,17,24]. Irrespective of gender, middle- to older-aged individuals are more likely to be heat intolerant compared to younger individuals [25]. While BSA/M ratio did not predict heat tolerance, BF% was associated with increased heart rate and skin temperature during heat stress [16,26]. Further, the risk of EHI was higher in obese military personnel compared to their counterparts who have BMI within the normal range [18,19,20]. Dehydration is another factor that increases the risk of EHI and military studies have reported higher incidences of EHI among dehydrated personnel [3,27,28]. Given that physical activities are often intense and prolonged while wearing protective clothing, heat storage and sweating increase and this may result in dehydration when sweat loss exceeds fluid intake [29]. Dehydration of one to two per cent body mass impairs performance and may reduce heat tolerance, while body mass loss of three per cent or more may exacerbate morbidity associated with EHI [30,31]. Although dehydration has been proven to be associated with EHI [3,27,28], the relationship between dehydration and heat tolerance among military personnel is still under-researched.

To date, several studies have reported heat tolerance outcomes using the HTT [12,13,24,32,33], but only a few have investigated the predictors of HTT performance among military populations, and these were conducted in the United States of America [16,17]. Furthermore, no study has been conducted in Australia to investigate the predictors of heat intolerance among members of the ADF. Given the career implications of heat intolerance for Defence Force members (including possible medical discharge), it is important to identify the predictive role of individual characteristics in heat intolerance.

Therefore, this study aimed to identify the individual characteristics (age, gender, aerobic capacity and body composition) that predict heat intolerance. We hypothesised that individuals who are heat intolerant would have a lower aerobic capacity, lower BSA/M ratio, higher dehydration levels, higher BMI and higher BF% compared to their heat-tolerant counterparts. Further, we hypothesised that older age, female gender, aerobic capacity, a previous history of EHS and the body composition factors would affect core temperature and heart rate responses during the HTT.

## 2. Materials and Methods

This study employed a quasi-experimental pre-test design.

### 2.1. Participants

Participants aged between 18 and 60 years (to reflect the demographic profile of the ADF) were recruited from the military (*n* = 16), university (*n* = 22) and athletic community (*n* = 14). Participants were contacted via phone calls or emails, and their medical history details obtained via a medical pre-screening form. All participants were provided written and verbal information about this study and written informed consent was obtained. Due to ethical issues and the possible career risk associated with heat intolerance, we could not recruit ADF members as control participants in this study. Only members (14 males and 2 females) who had been referred for the HTT after a known or suspected episode of EHS and volunteered for this study were recruited. Based on ADF policy [11], ADF members who have had a suspected or known episode of EHS are referred for the HTT (author MJC is a provider for heat tolerance testing to the ADF Australia-wide). The ADF members were tested at least 7 weeks or more after their EHS incident. The exact timing of the HTT following EHS could not be controlled, as it was dependent on referral. Details of the heat illness event including type and intensity of the activity, the month the event occurred and other predisposing risk factors prior to the event were obtained from the ADF members. The other participants comprised athletes (5 males and 9 females) and students and staff of the University (19 males and 3 females) who volunteered for this study. The athletes comprised 11 triathletes and four (4) single sport (swimming and running) athletes (median years of training: 4.2 years) who undertook an average of 4–7 training sessions per week of moderate to high intensity. The student and staff volunteers also comprised a physically active group who engaged in 3–4 sessions per week of low- to moderate-intensity exercise. The exclusion criteria were (1) pregnant or lactating women; (2) a history of hypertension, malignant hyperthermia, or diabetes; (3) undergoing treatment for a mental disorder; (4) undergoing treatment for anaemia; and (5) using glucose-lowering agents or prednisolone or beta-blockers. Participants were advised to avoid caffeine, alcohol, exhaustive exercise and heat exposure for 24 h before taking part in the heat tolerance test. This study was conducted using a similar methodology published by Lisman et al. [16].

### 2.2. Main Outcome Measure

Heat tolerance test outcomes:

The heat tolerance status was determined using the HTT performed in the morning at the same time of day for all participants to minimise the effects of circadian rhythms. The HTT procedure was based on a protocol published by Druyan et al. [13]. The HTT was conducted in a climate control chamber set to hot/dry conditions (40 °C, 40% relative humidity). Participants walked on a treadmill for 2 h at 5 km/h with a 2% incline. An individual was deemed heat intolerant if the core temperature was >38.5 °C, the heart rate (HR) was >150 bpm or when the core temperature failed to plateau (increase >0.45 °C during the 2nd hour of the HTT). The HTT was discontinued if the participants experienced nausea, headaches, weakness, dizziness or requested to discontinue the test. Participants were provided with water *ad libitum,* and the volume of water consumed was recorded for calculation of sweat rate.

Physiological measures during the heat tolerance test:

Participants were asked to arrive at the laboratory in a euhydrated state which was confirmed via urine sample with a urine specific gravity (USG) of <1.015 (Atago hand refractometer, Atago Co, Ltd., Itabashi, Tokyo, Japan). Participants whose USG were above 1.015 were provided with water to hydrate before the test commenced and their hydration state was confirmed by a second assessment of urine specific gravity. Resting HR (Polar T31 Coded Transmitter, Polar Electro Oy, Kempele, Finland) and blood pressure (Aneroid Sphygmomanometer Two-Handed, ALP-K2, Tokyo, Japan) were obtained before the heat tolerance test commenced. Rectal core temperature (RET-1 Rectal Probe, Physitemp Instruments, LLC, Clifton, NJ, USA) was assessed at rest and every 5 min during the heat tolerance test. Skin temperatures were obtained using wireless temperature logging iButtons (Thermochron, iButtonLink, LLC, Wisconson USA) from four sites (right upper chest, right mid-forearm, right mid-anterior thigh, right medial posterior calf located using anthropometric landmarks) for the measurement of the mean (averaged over the 2-h test duration) skin temperature (T _skin_). T_skin_ was calculated using the following equation [34]:

T _skin_ (°C) = 0.3 (T _chest_ + T _arm_) + 0.2 (T _thigh_ + T _leg_)

where T is the temperature at the different sites.

Temperature and HR measures were obtained at the start of the test and every 5 min throughout the exercise.

Other physiological measures, including the physiological strain index (PSI) and sweat rate, were calculated using standard methods. PSI was calculated using the equation derived by Moran et al. [35].

PSI = 5 (T _ret_ − T _re0_) * (39.5 − T _re0_)^−1^ + 5 (HR_t_ − HR_0_) * (180 − HR_0_)^−1^

where T _ret_ and HR_t_ were simultaneous measures of rectal temperature and HR taken at any time (5 min) during the exposure and T _re0_ and HR_0_ were the initial measurements.

The sweat rate was determined using the pre-exercise and post-exercise body weight (Tanita RD-545, Tanita Corporation, Tokyo, Japan), the volume of fluid ingested during the test and urine excreted. The sweat rate was calculated at the end of the heat tolerance test using the following equation [36]:

$$SR=\;\frac{\left(Pre\;exercise\;body\;weight-post\;exercise\;body\;weight\right)\;+\;\left(fluid\;ingested\;during\;exercise\right)-\left(urine\;excreted\;during\;exercise\right)}{Exercise\;time\;in\;hours}$$

where SR is sweat rate, weight in kg, fluid and urine in litres (L).

In addition, dehydration (% BM loss) was determined based on weight loss as a percentage of starting body mass (weight) [31]. Where BM (body mass) is weight in kg.

Perceptual measures during the heat tolerance test:

Perceptions of effort, thermal sensation and thermal comfort were assessed during the HTT. Ratings of perceived exertion (RPE) were assessed on a scale of 6–20 using Borg’s scale (6—no exertion, 20—maximal exertion) [37], and the thermal sensation scale (ThS) ratings ranged from 1 “unbearably cold” to 13 “unbearably hot [38]. Perceived level of comfort (ThC) with the environmental temperature was assessed using the thermal comfort rating scale, from 1—comfortable to 5—extremely uncomfortable [38].

### 2.3. Independent Measures

Body composition measures:

The height of the participants was obtained using a stadiometer (Seca 220, Hamburg, Germany) before the heat tolerance test commenced. Weight and body fat percentage (BF%) were determined using a body composition analyser (Tanita RD-545, Tanita Corporation, Tokyo, Japan). Other measures such as the body mass index (BMI), body surface area and body surface area to mass ratio were calculated using validated methods.

BMI was calculated using the equation proposed by Quetelet [39] =

$$\frac{body\;weight\;\left(kilogram\right)}{height^{2}\left(meters\right)}$$

The body surface area (BSA) was determined with the formula by Dubois and Dubois [40].

BSA (m^2^) = 0.0007184 * W^0.425^ * H^0.725^, where W is the weight and H is the height. The calculated BSA was used to determine the body surface area to mass ratio (BSA/M _ratio_).

VO_2_ max (Cardiorespiratory capacity test):

Participants performed a maximal ramp incremental test to determine the VO_2_max using a stationary cycle ergometer on the average within one week of the HTT (Excalibur Sport, Lode B.V, Groningen, The Netherlands) [41]. A three-minute warm-up was conducted before the test commenced. The initial workload was set at 100 W for males, and 50 W for females and the workload increased at a rate of 25 W min^−1^. The participants were instructed to maintain a cadence of at least 70 rpm. Participants were verbally encouraged to continue the test until exhaustion. Expired gases were collected and analysed using a calibrated metabolic cart (VIASYS Healthcare Inc, Conshohocken, Pennsylvania, Philadelphia, USA). HR was recorded every minute using a Polar heart rate monitor, and RPE was recorded every two (2) minutes using the Borg’s 6–20 rating scale [37]. VO_2_max was determined based on at least two of the following criteria: (1) no increase in the VO_2_ or HR with increasing exercise intensity; (2) a respiratory exchange ratio greater than 1.10; (3) an RPE greater than 17 on the Borg scale; and (4) a heart rate above of 90% of age-predicted maximum (220-age) [42,43].

### 2.4. Statistical Analyses

Statistical analyses were conducted using the Statistical Package of Social Sciences (SPSS Statistics, version 26, IBM Corp, Armonk, New York, USA). Heat tolerance status was analysed as a categorical dichotomous variable (heat tolerant versus heat intolerant) and as a continuous variable (final Tc and final HR during the heat tolerance test). The Shapiro–Wilk test was used to determine whether the data were normally distributed or not. Numerical data were presented as means and standard deviations, while categorical data were presented as frequencies and proportions. Where data were normally distributed (parametric), the independent sample *t*-test was used to analyse the mean differences in age, anthropometric, aerobic capacity and physiological responses between the groups. Where the data were non-parametric, a Mann–Whitney U test was used. A chi-square test of independence was used to analyse the relationship between EHS and heat tolerance status as well as gender differences between the groups. Pearson’s correlation was used to analyse the correlation between the heat tolerance outcomes (final Tc and HR), individual characteristics (age, BMI, BSA, BSA/M ratio, BF%, %BM loss and VO_2_max) and perceptual measures (thermal comfort, thermal sensation, RPE). Multiple linear regression analyses were run to predict heat intolerance (dependent variable) using anthropometric indices, aerobic capacity (independent variables), age, gender and history of EHS (covariates). The initial model included all independent variables which were examined to assess and determine their contribution to the dependent variable. Subsequently, predictors that did not make a significant statistical contribution to the model were removed from the model. Multicollinearity, homoscedasticity, unusual points, independence of errors, and normality residuals were all tested, and assumption conditions met. The level of significance was set at 0.05.

### 2.5. Ethics Approval

All participants gave their informed consent for inclusion before they participated in this study. This study was conducted following the Declaration of Helsinki and the National Health and Medical Research Council’s National Statement on Ethical Conduct in Human Research. Ethical approval was provided by the Departments of Defence and Veteran’s Affairs Human Research Committee (075-18) on 21 January 2019.

## 3. Results

This study comprised 52 participants (38 males and 14 females) with a mean age of 31.12 (SD = 11.59). The average height and weight of the participants were 176.9 ± 7.8 cm and 79.64 ± 13.23 kg, respectively. Overall, 56% (*n* = 29) were heat tolerant, while 44% (*n* = 23) of the participants met one or more of the criteria for heat intolerance. Nineteen heat-intolerant participants (83%) were males, while 4 (17%) were females. Nine participants (56%) with a previous history of exertional heat stroke were classified as heat intolerant. However, there were no significant age and gender differences between the heat-tolerant and -intolerant groups (Table 1). Similarly, there was no significant difference between having a previous history of EHS and heat tolerance. In contrast, significant differences in anthropometric measures between the groups were observed (Table 1). Heat-intolerant participants had significantly higher body weight (*p* = 0.007), higher BMI (*p* = 0.011), greater BF% (*p* = 0.034), and BSA (*p* = 0.017). Furthermore, the heat-intolerant participants had lower BSA/M _ratio_ (*p* = 0.005). The VO_2_max of the heat-intolerant participants was 23% significantly lower (39.2 mL/kg/min vs. 48.2 mL/kg/min) than the VO_2_max of the heat-tolerant participants (*p* = 0.001).

Participant characteristics by gender are provided in Table 2. All characteristics showed significantly higher values for males compared to females, except for BMI, VO_2_max and percentage dehydration (% BM loss).

Table 3 presents the differences in physiological and perceptual responses to the heat tolerance test between the heat-tolerant and -intolerant participants. There was no significant difference in baseline core temperature (T_c_), baseline heart rate (HR), sweat rate, percentage dehydration (% BM loss) and mean T _skin_ between the groups (Table 3). However, the heat-intolerant group had significantly higher final T_c_, final HR, and PSI (*p* < 0.001, respectively). In addition, the final RPE, final ThC and final ThS were significantly higher in the heat-intolerant group compared to the heat-tolerant group (Table 3).

Pearson’s correlation analysis showed that the final Tc was positively correlated with body fat% and BMI and negatively correlated with BSA/M _ratio_ and VO_2_max. Final HR showed a positive, but weak correlation with body fat% and a weak and moderate negative correlation with age and VO_2_max, respectively. PSI was positively correlated to BMI and negatively correlated to BSA/M _ratio_ and VO_2_max (Table 4).

The relationship between gender and each heat tolerance outcome was assessed, and the results showed that male participants had a significantly higher mean final Tc compared to their female counterparts (38.36 °C ± 0.43 vs. 38.00 °C ± 0.50; *t* = 2.59; *p* = 0.013). In addition, there was a significant difference in sweat rate, with males having a higher sweat rate than females (1.31 L/h vs. 1.13 L/h, Mann–Whitney U = 158.50; *p* = 0.027). However, gender had no effect on final heart rate or PSI.

The effect of a previous history of EHS on the heat tolerance test measures was investigated. Participants who had a previous heat stroke event had significantly higher final Tc compared to individuals who had no history of EHS (38.47 °C ± 0.30 vs. 38.17 °C ± 0.51; *t* = 2.69; df = 45.41; *p* = 0.010). A previous history of EHS had no significant effect on the other physiological responses to heat tolerance. Furthermore, details of the heat illness event were obtained from the ADF members. While all members identified physical activity as a risk factor (extrinsic) for EHS, basic fitness assessment exercises (sit-ups, push-ups and run) and pack marches were the most common activities (50% and 25%, respectively). Eleven (69%) participants had the heat illness event during the spring and summer months (high environmental temperatures), and of these participants, seven (7) were heat intolerant. The intrinsic factors reported were poor hydration (43.8%), poor sleep (25%) and prior illness (16.7%).

### Predictors of Heat Tolerance using Anthropometric and Capacity Measures

To identify the predictors of each heat tolerance outcome (final T_c_, and final HR) and PSI, a series of multiple linear regression analyses were conducted. Multiple regression analyses showed that BF%, VO_2_max and gender significantly predicted final Tc (F (3, 47) = 26.22, *p* < 0.001, adj R^2^ = 0.602. VO_2_max and age significantly predicted final HR (F (2, 48) = 23.26, *p* < 0.001, adj R^2^ = 0.471.

As shown in Table 5, there was a negative relationship between VO_2_max and final Tc such that for every unit increase in VO_2_max, final Tc decreased by 0.027 °C. In contrast, a unit increase in BF% was associated with a 0.020 °C in final Tc. The predicted final Tc for males was 0.424 °C higher than the final Tc for females. Similarly, negative correlations were observed between VO_2_max, age and final HR (Table 5). This implies that for every unit increase in VO_2_max and age, final HR decreased by 1.463 and 0.589 bpm, respectively. In addition, the multiple regression analysis showed that VO_2_max and gender predicted PSI (F (2, 46) = 23.46, *p* < 0.001, adj R^2^ = 0.467. For every unit increase in VO_2_max, PSI decreased by 0.090, and the PSI for males was 0.876 times higher than the PSI for females (Table 6).

## 4. Discussion

It is presumed that individuals who are heat intolerant have an increased risk of repeated exertional heat illness events and the HTT is used to determine return to duty to reduce the risk. While the HTT provides quantitative estimates of heat tolerance, there is limited evidence on the effect of aerobic capacity and body composition factors that determine heat tolerance. In this study, we estimated the rate of heat intolerance and investigated the factors that predicted performance in each heat tolerance outcome separately (final Tc and final HR). Aerobic capacity (VO_2_max), BMI, BSA/M _ratio_, and BF% were found to be associated with heat intolerance in our study. However, only aerobic capacity (VO_2_max), BF%, male gender and age contributed significantly and predicted final core temperature and final heart rate. Further, aerobic capacity and gender contributed to the physiological strain experienced during the exercise.

The rate of heat intolerance in our study was 44%. Previous studies have reported heat intolerance rates ranging from 25% to 47% [12,16,17,24,32,33]. Three of the studies were conducted only among military personnel with a previous history of EHS [24,32,33], one study included military controls [12], while the other two studies combined military personnel and other fit participants [16,17]. The varying heat intolerance rates may be explained by aerobic fitness. In most of the studies, the mean aerobic fitness reported for the participants were higher than the average aerobic fitness for both groups in our study [16,17,32].

The influence of aerobic capacity on overall performance in the heat tolerance test including core temperature, heart rate and PSI identified in this study is consistent with previous studies that revealed that aerobic capacity predicted heat tolerance in fit populations and military personnel [16,44]. In our study, the heat-tolerant participants had a higher aerobic capacity compared to the heat-intolerant group who were less fit. According to the evidence, aerobically fit individuals have improved heat tolerance compared to unfit individuals [45]. Aerobic capacity confers a protective mechanism that is similar to the physiologic adaptations gained from heat acclimatization and includes increases in stroke volume and blood volume [45]. These physiological adaptations reduce the cardiovascular strain associated with heat stress [45]. However, unfit or less fit individuals have lower blood volume and stroke volume and tend to experience greater circulatory strain at any given core temperature during heat stress [46,47]. The greater circulatory/cardiovascular strain among the unfit was reflected in the higher exercise heart rate and reported PSI in our study. Previous studies have reported an inverse relationship between aerobic capacity and heart rate during the heat stress test. These studies suggest that high aerobic capacity diminishes the physiological strain during exercise [26,45,48].

While weight, BSA, BMI, BF% and BSA/M _ratio_ were found to be associated with heat intolerance, BF% was the only anthropometric factor contributing to the prediction of final core temperature. The significant association between the other anthropometric measures (BSA, BMI, and BSA/M _ratio_) and heat intolerance may be because the measures were assessing a similar construct in relation to body weight. Our finding that an increase in BF% results in an increase in final core temperature aligns with existing literature due to the lower heat capacity of adipose tissue [47]. Previous studies have reported that individuals with a higher BF% have higher core temperature and heart rate and are at increased risk of EHI [18,21,49]. It is also important to note that the effect of body fat% on final core temperature was in association with aerobic capacity, which was negatively correlated with body fat% in this study- thus highlighting the role of aerobic capacity in heat intolerance.

Other factors such as gender and age contributed to the observed variation in final core temperature, heart rate and PSI, respectively. Male participants had an increased risk of higher core temperature than their female counterparts in this study. This finding is in contrast to previous studies conducted in military populations which have reported that females were more likely to have either a higher core temperature [16] or higher final heart rate during HTT [17]. Evidence suggests that gender differences in thermoregulation during exercise in the heat are negligible once aerobic fitness is considered [50]. While there was no significant gender difference in aerobic capacity, males had a higher physiological strain compared to females in the current study. According to Moran et al., a lower physiological strain is a reflection of a higher aerobic capacity [51]. However, it is important to note that the number of males in this current study was almost thrice the number of females. Further, the proportion of heat-intolerant males were greater than the heat-intolerant females, likely due to the higher proportion of ADF males referred for heat tolerance testing following EHS. Thus, the predicted higher core temperature and physiological strain among males might be a reflection of the higher number of males in this study compared to females.

Another demographic variable that was found to be a predictor of HR (as a criterion for heat intolerance) was age. The findings of the current study suggest final HR was lower with advancing age. In this study, 50% of the participants who were aged 40 years and older were athletes with high aerobic capacity. The lower HR in the older age group may be explained by the fitness levels of the active older participants. This finding agrees with existing literature which reported either no difference in final HR or a lower HR among active older individuals when matched for aerobic fitness with younger people while exercising in hot environmental conditions [25]. Pandolf et al. showed that aerobically fit middle-aged men had lower HR compared to younger men when matched for fitness levels [52]. According to Smolander et al., there was no difference in HR between younger and middle-aged participants during heat stress. The authors concluded that physical activity habits and aerobic fitness are important determinants of heat tolerance in older individuals [53]. In contrast, a study conducted among military personnel reported that age had no influence on the final heart rate during the HTT [16]. However, it is important to note that we had an older and highly active age group (oldest—57 years) compared to the age group (oldest—45 years) in the above study [16]. The finding of our study buttresses the effect of aerobic fitness on heat tolerance. Future military-related research on heat tolerance could focus on examining the effect of matching older aerobically fit military personnel with younger personnel. Finally, there was no significant difference in the level of dehydration between the heat-tolerant and -intolerant participants, implying that it is not a predictor of heat intolerance in this study. Previous studies have indicated that dehydration affects performance and may increase the risk of EHI [31]. The contrasting finding in this study may be associated with provision of *ad libitum* water that resulted in mild %dehydration in the study population.

### 4.1. Strengths and Limitations

This paper adds to the existing literature by identifying the predictors of heat intolerance among military and non-military populations. However, it is important to highlight the limitations of this study. First, we could not establish causal relationships due to the narrow range of observed values for the independent variables. For example, it was necessary to recruit fit participants to this study to reflect the military population, which resulted in a small range of values for the VO_2_max. Second, the use of a cycle ergometer for the estimation of aerobic capacity may have caused a reduction in the aerobic capacity levels among the participants. Compared to a treadmill protocol, participants are more likely to cease exercise due to intolerable leg discomfort associated with cycling, especially among those who do not cycle or are unfit [54]. Third, we estimated %BF using Tanita Scale, which is reported to have <1% error on repeated measurements. However, body impedance varies among different people and influences the accuracy of bioelectrical impedance analysis [55]. Fourth, testing spanned the summer and winter periods in North Queensland, Australia, which may have affected acclimatisation to heat. Nonetheless, it is important to note that in North Queensland, Australia, the winter periods are warm, and the summers are hot and humid. Fifth, we acknowledge that the non-military participants included in this study may not have had similar levels of exposure to hot environmental conditions and tasks like the military personnel. However, we ensured that the participants were similar to the military personnel with regards to age, gender and aerobic capacity level.

### 4.2. Implications for Policy and Future Research

Military personnel are involved in operations that may expose them to extreme environmental temperature in addition to rigorous physical activities which can create un-compensable heat stress and may cause heat illnesses. Nonetheless, the findings of our study highlight the importance of maintaining a high aerobic capacity and low BF% to aid thermotolerance and to reduce the physiological strain experienced during exercise. Therefore, aerobically fit personnel will be able to perform better in the heat for longer durations than unfit personnel. It is important for military planners to ensure that the training exercises/deployments undertaken by personnel are matched to their capacity level to reduce the risk of EHI. However, this does not negate the importance of ensuring aerobic capacity levels are increased to limit incidences of EHI. Furthermore, military personnel need to maintain low BF% levels to reduce the risk of heat intolerance and EHI. While our study was focused on military populations, our findings could provide some directions into future research on the HTT for other active populations.

Although we did not investigate the predictive value of the HTT, it is worth mentioning that based on the HTT criteria, 56% of participants who had a history of EHS (ADF members) were heat intolerant. While the HTT cannot determine the reoccurrence of EHS, it is reflective of the heat tolerance status at the time of testing. This implies that they may not have fully recovered from the heat illness event before presenting for the HTT. The heat intolerance outcome for this group of ADF members indicates there might be a possible interplay between physiological responses and other intrinsic factors (such as haematological, biochemical and genetic biomarkers). While clinical biomarkers of EHS have been reported in the literature [56], little is known about their role in relation to heat intolerance. Based on the limitations (sensitivity and specificity) of the HTT, these biomarkers could be included in the decision-making process, in addition to the HTT protocol, when determining return to duty. It has previously been suggested that the decision-making process should involve the use of as many reliable tools as possible, including clinical biomarkers, patient history and the HTT [15]. It is evident that possible future research is required to elucidate the function of these clinical biomarkers in the recovery process, which may potentially aid the development of a more reliable protocol.

## 5. Conclusions

The current study has demonstrated that individual characteristics, including aerobic capacity (VO_2_max), BF%, age and gender are predictors of heat intolerance among military and non-military populations during a HTT. Given that heat intolerance may precede or accompany EHS, the factors that influence heat tolerance should be accurately identified to reduce the risk of EHI. Furthermore, our study has raised questions about the need for other indicators of heat intolerance such as clinical biomarkers to be identified and used in conjunction with the HTT protocol and possibly for the development of a potential reliable protocol.

## Figures and Tables

**Table 1:** Physical and anthropometric characteristics and heat tolerance status of the participants.

Characteristics	Heat Tolerant	Heat Intolerant	Test Statistic	*p*-Value
	Mean ± SD	Mean ± SD	*t*-test	
Height (cm)	176.2 ± 8.7	177.7 ± 6.4	−0.688	0.495
Weight (kg)	75.5 ± 12.10	85.3 ± 12.8	−2.836	0.007
BMI (kg/m^2^)	24.4 ± 3.3	27.0 ± 3.9	−2.630	0.011
BSA (m^2^)	1.9 ± 0.2	2.0 ± 0.2	−2.470	0.017
BSA/M _ratio_ (cm^−2^/kg)	256.5 ± 20.0	240.4 ± 19.5	2.913	0.005
VO_2_max (mL/kg/min)	48.2 ± 10.5	39.2 ± 6.4	3.532	0.001
Body fat (%)	20.2 ± 5.9	24.1 ± 7.0	−2.183	0.034
	Median (IQR)	Median (IQR)	Mann–Whitney U	
Age	31.0 (16)	26.5 (17)	279.00	0.349
	*N* (%)	*N* (%)	Chi Square	
Previous history of EHS	7 (43.8)	9 (56.3)	1.353	0.245
No history of EHS	22 (61.1)	14 (38.9)		
Female	10 (71.4)	4 (28.6)	1.904	0.165
Male	19 (50.0)	19 (50.0)		

BMI, body mass index; BSA/M ratio, body surface area to mass ratio; EHS; exertional heat stroke; IQR, interquartile range.

**Table 2:** Participant characteristics by gender.

Characteristics	Male	Female	Test Statistic	*p*-Value
	Mean ± SD	Mean ± SD	*t*-test	
Height (cm)	180.5 ± 4.8	166.9 ± 5.0	8.977	<0.001
Weight (kg)	84.2 ± 11.5	67.4 ± 9.4	4.882	<0.001
BMI (kg/m^2^)	25.9 ± 4.1	24.2 ± 2.6	1.463	0.150
BSA (m^2^)	2.0 ± 0.1	1.8 ± 0.1	7.689	<0.001
BSA/M _ratio_ (cm^−2^/kg)	2.4 ± 0.2	2.6 ± 0.2	−2.870	0.006
VO_2_max (mL/kg/min)	43.8 ± 10.2	45.6 ± 9.5	−0.579	0.565
Body fat (%)	2.4 ± 0.2	2.6 ± 0.2	−2.395	0.028
%BM loss (kg)	1.54 ± 1.36	1.20 ± 0.64	0.894	0.376

**Table 3:** Differences in physiological and perceptual responses to the heat tolerance test between heat-tolerant and heat-intolerant participants.

Characteristics	Heat Tolerant	Heat Intolerant	Test Statistic	*p*-Value
	Mean ± SD	Mean ± SD	*t*-test	
Baseline T_C_ (°C)	36.87 ± 0.29	37.02 ± 0.29	−1.811	0.076
Final T_C_ (°C)	37.96 ± 0.37	38.67 ± 0.22	−8.051	<0.001
Baseline HR (bpm)	65 ± 11	70 ± 10	−1.631	0.109
Final HR (bpm)	122 ± 18	154 ± 15	−7.010	<0.001
PSI	3.5 ± 1.0	5.6 ± 1.3	−6.404	<0.001
	Median (IQR)	Median (IQR)	Mann–Whitney U	
Mean T_skin_ (°C)	35.61 (0.93)	36.08 (0.84)	214.00	0.053
Final RPE	11.00 (3.0)	13 (6.0)	200.50	0.016
Final ThC	2.25 (3.5)	3.5 (1.5)	160.50	0.001
Final ThS	10.00 (1.0)	11.00 (2.3)	182.00	0.005
SR (L/hr)	1.18 (0.28)	1.32 (0.80)	284.500	0.399
%BM loss (kg)	1.38 ± 0.82	1.53 ± 1.59	−0.463	0.645

Tc, core temperature; HR, heart rate; PSI, physiological strain index; RPE, rate of perceived exertion; ThC, thermal comfort; ThS, thermal sensation; SR; sweat rate; %BM loss, %body mass loss (% dehydration); IQR, interquartile range.

**Table 4:** Pearson’s correlation coefficients between anthropometric, body composition and fitness measures and heat tolerance test outcomes.

Characteristics	Final T_C_	Final HR	PSI
Age	−0.13	−0.32 *	−0.13
Anthropometric and body composition measures			
BMI	0.49 **	0.25	0.35 *
BSA/M _ratio_	−0.54 **	−0.27	−0.38 **
Body fat%	0.34 *	0.29 *	0.25
%BM loss	−0.03	0.06	0.14
Fitness measure			
VO_2_max	−0.70 **	−0.64 **	−0.62 **

** *p* < 0.01; * *p* < 0.05; BMI, body mass index; BSA ratio, body surface area to mass ratio; %BM loss, %body mass loss (% dehydration); Tc, core temperature; HR, heart rate; PSI, physiological strain index.

**Table 5:** Multiple regression analysis showing the factors that predict heat tolerance outcomes (final Tc and final HR).

	Variables	R^2^	Adj R^2^	ꞵ	SE_B_	B	t	*p*-Value	VIF	Comment
Final Tc	Constant	0.626	0.602	38.737	0.362		106.976	<0.001		Non-collinearity
	BF%			0.020	0.008	0.272	2.555	0.014	1.427	Non-collinearity
	Gender (Male)			0.424	0.105	0.400	4.028	<0.001	1.237	Non-collinearity
	VO_2_max			−0.027	0.005	−0.566	−5.692	<0.001	1.242	Non-collinearity
Final HR	Constant	0.492	0.471	218.435	12.475		17.509	<0.001		Non-collinearity
	VO_2_max			−1.463	0.240	−0.628	−6.099	<0.001	1.001	Non collinearity
	Age			−0.589	0.205	−0.296	−2.872	0.006	1.001	Non collinearity

β, unstandardized coefficient; SE_B_, residual mean square errors associated with B; B, standardized coefficient; *p*, significance level; VIF, variance inflation factor.

**Table 6:** Multiple regression analysis showing the factors that predict PSI.

	Variables	R^2^	Adj R^2^	ꞵ	SE	B	t	*p*-Value	VIF	Comment
PSI	Constant	0.489	0.467	7.677	0.739		10.391	<0.001		Non-collinearity
	Gender (Male)			0.876	0.343	0.269	2.551	0.014	1.002	Non-collinearity
	VO_2_max			−0.090	0.015	−0.633	−5.999	<0.001	1.002	Non-collinearity

β, unstandardized coefficient; SE_B_, residual mean square errors associated with B; B, standardized coefficient; *p*, significance level; VIF, variance inflation factor.

## Data Availability

The data analysed and presented in this study are included in in this published article.

## Abbreviations

ADF	Australian Defence Force
BF%	Body fat percent
BMI	Body mass index
BSA/M ratio	Basal surface area to mass ratio
EHS	Exertional heat stroke
EHI	Exertional heat illness
HR	Heart rate
HTT	Heat tolerance test
PSI	Physiological strain index
RPE	Ratings of perceived exertion
Tc	Core temperature
ThC	Thermal comfort
Ths	Thermal sensation scale
USG	Urine specific gravity
